# Le choriocarciome endometrial non gestationnel chez une femme ménopausée et nullipare: à propos d'un cas

**DOI:** 10.11604/pamj.2014.17.278.3476

**Published:** 2014-04-14

**Authors:** Ali Sbai, Asmae Ouabdelmoumene, Farid Naciri, Mohammed Elhfid, Loubna Mezouar

**Affiliations:** 1 Centre régional d'oncologie Hassan-2-Oujda, Maroc; 2 Faculté de Médecine et de Pharmacie, Université Mohammed Premier,Oujda, Maroc

**Keywords:** Non gestational endometrial choriocarcioma, femme ménopausée, béta hCG plasmatiques, stade précoce, Choriocarciome endometrial non gestationnel, menopausal women, plamatic beta hCG, early stage

## Abstract

Le choriocarcinome est une tumeur trophoblastique hautement maligne. La plupart des cas de choriocarcinome sont intra utérins et d'origine gestationnelle. Le choriocarcinome chez les femmes ménopausées est extrêmement rare. Nous rapportons une observation originale d'une femme ménopausée et nullipare présentant un choriocarciome endometrial non gestationnel survenant 17 ans après sa ménopause. Il n'existe pas de guidelines bien définis pour le traitement des choriocarcinomes en post ménopause. Leur pronostic est très facheux, pourtant il paraît que le stade précoce de la maladie ainsi que les béta hCG plasmatiques négatives seraient à l'origine de ces survies: globale et sans progression relativement longues chez notre patiente (32 mois).

## Introduction

Le choriocarcinome est une tumeur trophoblastique hautement maligne composée de deux types de cellules: syncitio-trophoblastiques et cytotrophoblastiques [1].la plupart des cas de choriocarcinome sont intra utérins et d′origine gestationnelles.

Les choriocarciomes non gestationnels dériveraient des cellules germinales multipotentes se développant le plus souvent dans les gonades. Le choriocarcinome est une complication rare de la grossesse qui se développe souvent à partir d′une grossesse môlaire antérieure ou plus rarement d′une grossesse non môlaire survenant dans l′année suivant la dernière grossesse [2].

Le choriocarcinome chez les femmes ménopausées est très rare toutefois quelques cas de choriocarcinomes se développant après une longue période de latence à partir de la dernière grossesse ont été rapportés [2, 3].nous rapportons une observation originale d′une femme ménopausée et nullipare présentant un choriocarciome endometrial non gestationnel survenant 17 ans après sa ménopause.

## Patient et observation

**Figure 1:**
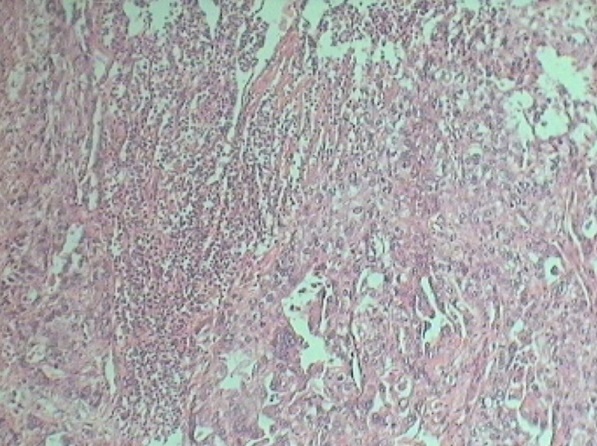
Coupe histologique infiltration myometriale: grossissement x 100

**Figure 2:**
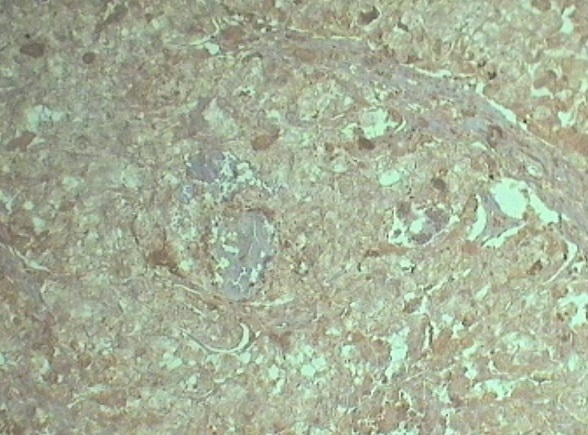
Immunohistochimie avec beta hcg positivité franche: grossissement x 100

**Figure 3:**
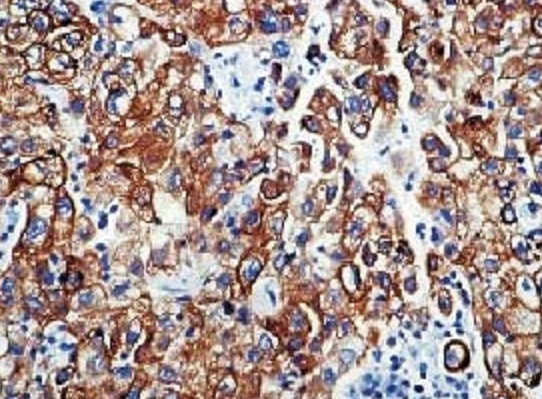
Choriocarcinome IHC AC anti pancytokeratine

Patiente âgée de 74 ans, ménopausée il y a 17 ans, n′a jamais été mariée et n′ayant eu aucune grossesse qui rapporte la survenue depuis l′année 2010 de métrorragies de faible abondance sans aucun signe associé, elle s′est négligée pendant un an jusqu′en 2011 ou elle a consulté un gynécologue pour la première fois,une échographie pelvienne avait montré la présence d′une masse utérine d′allure tissulaire, elle a bénéficié d′une hystérectomie totale avec annexectomie bilatérale. L′étude histologique a permis de montrer la présence d′une tumeur endométriale sous forme de prolifération de cellules cyto et syncitio-trophoblastiques, agencées en plages compactes et massives s′insinuant entre les fibres musculaires lisses avec des emboles trophoblastiques intra vasculaires suspectant fortement un choriocarcinome (Figure 1). L′étude immunohistochimique était positive avec les béta hCG (Figure 2), cytokératines (clone AE1/AE3) (Figure 3) ainsi que les anti-PALP (clone NB-10,cell Marque). Les dosages sanguins de l′AFP et des Ca-125 étaient normaux, les béta hCG sanguines étaient négatives. Un bilan d′extension (radiographie pulmonaire et échographie abdominopelvienne) s′est révélé normal.vu la négativité des béta hCG plasmatiques et l′absence de métastases à distance on a opté pour une surveillance seule avec dosage des béta hCG tous les 3 mois et bilan radiologique tous les ans. Après 30 mois de recul la patiente est toujours vivante, ses béta hCG plasmatiques sont restés toujours négatives et son bilan radiologique est strictement normal.

## Discussion

Le choriocarcinome peut être divisé en deux types: gestationnel et non gestationnel.les choriocarcinomes surviennent généralement chez les femmes en âge de reproduction, souvent dans la première année suivant une grossesse môlaire ou non môlaire. Les choriocarciomes non gestationnels peuvent se développer à partir des cellules germinales ou d′une différenciation trophoblastique se produisant dans un carcinome endometrial.les tumeurs germinales extra ovariennes (choriocarcinomes y compris) peuvent se développer à partir de cellules germinales qui ont échoué à compléter leur migration vers les gonades [4]. Cependant les choriocarcinomes provenant des cellules germinales et se développant dans le tractus génital féminin chez les femmes ménopausées avec des ovaires normaux à la TDM sont extrêmement rares [5, 6]. L’ étude immunohistochimique est d′un apport considérable pour le diagnostic différentiel du choriocarcinome, ainsi une positivité diffuse avec les béta hCG confirme le diagnostic de choriocarcinome. Les anticorps AE1/AE3 sont aussi souvent positifs dans les choriocarcinomes. Les cytokératines sont souvent exprimés dans les cellules trophoblastiques car ces dernières sont dérivées des cellules épithéliales [7, 8].les taux sériques de l’ AFP des Ca-125 et des béta hCG sont aussi utilisés dans le diagnostic différentiel des choriocarcinomes. Généralement les taux de l′AFP et des Ca-125 sont élevés respectivement dans les tumeurs germinales non séminomateuses et dans les carcinomes ovariens. Dans notre cas l′étude immunohistochimique était positive avec les béta hCG, AE1etAE3, les taux sériques de l′AFP et des Ca-125 étaient normaux et celui des béta hCG était négatif. Selon la classification de la FIGO des GTT (gestational trophoblastic tumors) [9]: notre patiente était classée: stade I (tumeur limitée à l’ uterus, absence de métastases à distance, béta hCG plasmatique négatives). Par contre elle ne pouvait être classée selon le (modified WHO prognostic scoring system adapted by FIGO) [9] en raison de l′absence de grossesse antérieure. Il n′existe pas de guidelines bien définis pour le traitement des choriocarcinomes en post ménopause: dans les stades avancés les études antérieures ont suggéré l′efficacité d′une chimiothérapie selon le protocole (EMA/CO): etopside, methotrexate, dactinomycine suivis de cyclophosfamide, vincristine. Dans notre cas nous avons opté pour une surveillance trimestrielle rigoureuse, notre patiente est restée vivante jusqu′à aujourd′hui sans aucune rechute ni biologique ni radiologique.

## Conclusion

Au meilleur de notre connaissance c'est le premier cas de choriocarcinome utérin non gestationnel chez une femme nullipare et ménopausée depuis 17 ans. Les choriocarcinomes sont extrêmement rares, leur pronostic est très facheux, pourtant il paraît que le stade précoce de la maladie ainsi que les béta hCG plasmatiques négatives seraient à l'origine de ces survies: globale et sans progression relativement longues (32 mois) chez notre patiente.

## References

[1] Nisarg R Desai, Shilip Gupta, Qun Dai (2010). Choriocarcinoma in a 73-year-old woman: a case report and review of the literature. J Med Case Rep..

[2] O'Neil CJ, Houghton F, Clarke J, McCluggage WG (2008). Uterine gestational choriocarcinoma developing after a long period in a postmenopausal woman:the value of DNA polymorphism studies. Int J Surg Pathol..

[3] Sonobe H, Taguchi K, Ogawa K, Yoshioka T (1976). Latent vaginal choriocarcinoma in a postmenopausal woman. Acta Pathol Jp..

[4] Weiss S, Amit A, Schwartz MR, Kaplan AL (2001). Primary choriocarcinoma of the vulva. Int J Gynecol Cancer..

[5] Fisher RA, Savage PM, Mac Dermott C, Hook J, Sebire NJ, Lindsay I, Seckl MJ (2007). The impact of molecular genetic diagnosis on the management of women with hCG-producing malignancies. Gynecol Oncol..

[6] Mukherjee U, Thakur V, Katiyar D, Goyal HK, Pendharkar D (2006). Uterine choriocarcinoma in a postmenopausal woman. Med Oncol..

[7] Ulbright TM (2005). Germ cell tumors of the gonads: a selective review emphasizing problems in differential diagnosis, newly appreciated, and controversial issues. Mod Pathol..

[8] Stiemer B, Graf R, Neudeck H, Hildebrandt R, Hopp H, Weitzel HK (1995). Antibodies to cytokeratins bind to epitopes in human uterine smooth muscle cells in normal and pathological pregnancies. Histopathology..

[9] National Cancer Institute (2013). Gestational Trophoblastic Disease Treatment (PDQ^®^), health professional: Stage Information for Gestational Trophoblastic Disease.

